# Integrierte Diagnostik antimikrobieller Resistenzen: Quantitative, qualitative und KI-gestützte Ansätze

**DOI:** 10.1007/s00103-026-04220-y

**Published:** 2026-03-20

**Authors:** Benjamin Berinson, Moritz Hentschke, Holger Rohde

**Affiliations:** 1 Institut für Medizinische Mikrobiologie, Virologie und Hygiene, Universitätsklinikum Hamburg-Eppendorf, Martinistr. 52, 20246 Hamburg, Deutschland; 2 Medilys Laborgesellschaft mbH, Hamburg, Deutschland

**Keywords:** Phänotypische Empfindlichkeitstestung, Beschleunigte Diagnostik, Molekulare Detektion, Ganzgenomsequenzierung, Künstliche Intelligenz, Phenotypic susceptibility testing, Rapid diagnostics, Molecular detection, Whole genome sequencing, Artificial intelligence

## Abstract

Die schnelle, weltweite Zunahme antimikrobieller Resistenzen erschwert die Behandlung lebensbedrohlicher Infektionen und erfordert schnelle, verlässliche Verfahren zur Antibiotikaempfindlichkeitsprüfung (AST). Während phänotypische Methoden wie Bouillondilution, Agardiffusion, Gradientendiffusion sowie automatisierte Systeme weiterhin den diagnostischen Standard darstellen, sind sie durch lange Turnaround-Zeiten limitiert. Beschleunigte phänotypische AST-Verfahren (RAST) verkürzen die Zeit bis zu ersten Ergebnissen auf 4–8 h und ermöglichen eine frühere Optimierung antiinfektiver Therapien, wenngleich ihr klinischer Nutzen bislang nicht abschließend belegt und ihr Einsatz auf validierte Erreger und Substanzen begrenzt ist.

Parallel erlauben molekulare Methoden wie PCR, isothermale Amplifikation und zunehmend auch Gesamtgenomsequenzierung den schnellen Nachweis zentraler Resistenzdeterminanten (zum Beispiel *mecA/C, vanA/B*, Extended-spectrum-Beta-Laktamasen (ESBL), Carbapenemasegene) und unterstützen so insbesondere die Aufarbeitung positiver Blutkulturen sowie Überwachungsuntersuchungen. Ihre Aussagekraft ist bei grampositiven Erregern hoch, bei gramnegativen jedoch durch die Vielfalt resistenzvermittelnder Mechanismen limitiert. Künstliche Intelligenz (KI) bietet zusätzliche Potenziale in der automatisierten Interpretation phänotypischer Tests, in der Analyse komplexer Genomdaten und in massenspektrometrischen Modellen zur Resistenzvorhersage, steht aber vor Herausforderungen hinsichtlich Standardisierung, Generalisierbarkeit und Datenqualität.

Insgesamt ergänzen neue RAST-, molekulare und KI-gestützte Ansätze die klassischen Methoden sinnvoll, ersetzen sie jedoch nicht. Ihr klinischer Nutzen entfaltet sich nur bei gezieltem Einsatz und Einbettung in funktionierende Antibiotic- und Diagnostic-Stewardship-Strukturen.

## Abstract (en)

The rapid global increase in antimicrobial resistance complicates the treatment of life-threatening infections and makes fast, reliable antimicrobial susceptibility testing (AST) essential. While phenotypic methods such as broth dilution, agar diffusion, gradient diffusion and automated systems remain the diagnostic standard, they are limited by long turnaround times. Rapid phenotypic AST (RAST) approaches shorten the time to first results to 4 to 8 h and allow earlier optimisation of anti-infective therapy, although their clinical benefit has not yet been conclusively demonstrated and their use is restricted to validated pathogens and substances.

In parallel, molecular methods such as PCR, isothermal amplification and, increasingly, whole-genome sequencing enable rapid detection of key resistance determinants (e.g., *mecA/C*, *vanA/B*, extended-spectrum beta-lactamases [ESBL] and carbapenemase genes), thereby particularly supporting the workup of positive blood cultures and surveillance investigations. Their predictive value is high for Gram-positive pathogens but limited for Gram-negative organisms due to the diversity of resistance mechanisms. Artificial intelligence (AI) offers additional potential for automated interpretation of phenotypic tests, analysis of complex genomic data and mass-spectrometry-based resistance prediction models, but faces challenges regarding standardisation, generalisability and data quality.

Overall, novel RAST, molecular and AI-supported approaches usefully complement but do not replace classical methods. Their clinical impact depends on targeted implementation and integration into effective antibiotic and diagnostic stewardship structures.

## Einleitung

Die weltweit zunehmende Prävalenz von Infektionen durch multiresistente Erreger stellt eine erhebliche klinische Herausforderung dar. Besonders bei schweren Infektionen und Sepsis ist antimikrobielle Resistenz von großer Bedeutung, da Verzögerungen der wirksamen Therapie deutlich mit einer klinischen Verschlechterung und einer erhöhten Mortalität assoziiert sind [1–3]. Eine schnelle Identifizierung des verursachenden Pathogens und des entsprechenden Resistenzprofils ist daher entscheidend für das erfolgreiche Management dieser Patienten [4].

Der häufige Einsatz breit wirksamer Antibiotika ist ein wesentlicher Mechanismus, der die Ausbreitung von antibiotikaresistenten Erregern fördert. Dieser ungeeignete Einsatz von Antiinfektiva soll durch die Implementierung sogenannter Antimicrobial-Stewardship-(ABS-)Programme unterbunden werden. ABS-Interventionen, vor allem Deeskalationsbemühungen, setzen verlässliche Erkenntnisse zur Antibiotikaempfindlichkeit klinischer Isolate voraus.

Im Folgenden werden die wichtigsten Methoden zur Prüfung der Antibiotikaempfindlichkeit sowie deren gezielter Einsatz und Implementierung im klinischen Alltag dargestellt. Dabei wird u. a. auf unterschiedliche phänotypische Methoden der Empfindlichkeitsprüfung, den Nutzen und die Limitierungen molekularer Nachweisverfahren sowie den möglichen Einsatz von künstlicher Intelligenz (KI) bei der Erkennung bakterieller Resistenzen eingegangen.

## Phänotypische Verfahren zur Resistenztestung

Phänotypische Resistenztestungsverfahren prüfen, ob ein bestimmtes Isolat in Anwesenheit einer definierten Konzentration eines Antibiotikums wachsen kann. Verfahren, die eine fixe Menge/Konzentration eines Antibiotikums einsetzen, ermöglichen nur eine kategoriale Bestimmung der Empfindlichkeit in sensibel (S), sensibel bei erhöhter Dosierung des Antibiotikums (I) und resistent (R). Verfahren, die bakterielles Wachstum bei unterschiedlichen Konzentrationen untersuchen, erlauben hingegen eine Bestimmung der minimalen Hemmkonzentration (MHK). Die MHK bezeichnet die niedrigste Konzentration eines Antibiotikums, bei welcher keine Vermehrung des Bakteriums mehr möglich ist, und scheint die wesentliche Kennzahl für die klinische Wirksamkeit eines Antibiotikums zu sein [5].

Die phänotypische Resistenztestung ist derzeit noch unverzichtbar für die Bestimmung der klinischen Wirksamkeit eines Antibiotikums. Doch die unterschiedlichen Verfahren der phänotypischen Empfindlichkeitstestung (AST) weisen Probleme auf.

So können die Ergebnisse bei Wiederholungsuntersuchungen erheblich schwanken und abweichende Test-zu-Test-Ergebnisse von 1–2 Verdünnungsstufen in der MHK bei der Untersuchung desselben Isolats gelten als normal. Dies kann im Einzelfall jedoch zu einer unterschiedlichen kategorialen Zuordnung (S/I/R) führen [6]. Faktoren, die Einfluss auf das Ergebnis der Untersuchung haben können, umfassen unter anderem Alter der Kultur des Isolats, Inokulum, verwendetes Medium der Ausgangskultur, verwendetes Medium zur AST, Temperatur und Dauer der Bebrütung. Daher hat das European Committee on Antimicrobial Susceptibility Testing (EUCAST) dezidierte technische Anleitungen für die Durchführung einer AST veröffentlicht. Nur Testungen unter den darin beschriebenen Bedingungen sind als valide zu betrachten (EUCAST: Disk Diffusion and Quality Control^1^). Trotz eines hohen Grades an technischer Standardisierung lassen sich Schwankungen jedoch nicht vollständig vermeiden. Diese Tatsache muss bei der Interpretation der Ergebnisse berücksichtigt werden [7].

Des Weiteren ist ein generelles Problem aller konventionellen AST-Verfahren die lange Dauer bis zum Vorliegen von klinisch nutzbaren Ergebnissen. Typische Inkubationsdauern betragen 16–20 h [8]. Dies führt dazu, dass Patienten gegebenenfalls über einen kritisch langen Zeitraum mit einer unwirksamen Therapie behandelt werden.

### Bouillondilution

Für das Bouillondilutionsverfahren werden definierte Inokula des zu testenden Erregers in eine Reihe von Flüssigkulturen gleichen Volumens eingebracht, welche sich jeweils verdoppelnde Konzentrationen eines bestimmten Antibiotikums enthalten [9, 10]. Die niedrigste Konzentration eines Antibiotikums, welche im entsprechenden Röhrchen makroskopisch sichtbares kulturelles Wachstum oder den Farbumschlag eines Indikators hemmt, kennzeichnet die MHK. Die Bouillondilution ist genormt und bildet das Referenzverfahren des EUCAST. An diesem Verfahren werden andere AST-Verfahren validiert.

Dennoch können auch hierbei Schwierigkeiten in der Interpretation auftreten. So bilden sich bei einigen Substanzen (zum Beispiel Cefiderocol) keine eindeutigen MHK-Grenzwerte, sondern das Wachstum wird über mehrere Verdünnungsstufen graduell weniger [11]. Weiterhin kommt es vor, dass bewachsene Röhrchen von unbewachsenen flankiert werden (und umgekehrt), sodass eine MHK nicht bestimmt werden kann. Des Weiteren ist das Verfahren recht aufwendig, sodass es nur bei gezielten Fragestellungen (wie etwa der MHK-Bestimmung von Colistin) im Routinelabor zum Einsatz kommt.

### Agardiffusion

Bei der Agardiffusion werden Festmedien mit einer Bakteriensuspension des zu testenden Pathogens beimpft. Auf das Medium werden Filterpapierplättchen gelegt, die mit definierten Antibiotikamengen beschickt sind. Das Antibiotikum diffundiert anschließend radial in das Medium, wobei die Konzentration mit zunehmendem Abstand vom Plättchen abnimmt. Bei Empfindlichkeit entsteht um das Plättchen eine Zone – der sogenannte Hemmhof –, in der das Wachstum des Isolats unterdrückt ist. Der Durchmesser dieses Hemmhofes kann unter Anwendung der EUCAST-Breakpoints zur kategorialen Zuordnung in S, I oder R herangezogen werden. Die Festlegung der jeweiligen Hemmhofgrenzen, die je nach Erreger und Antibiotikum variieren, basiert auf einer Korrelation zwischen MHK-Werten und Hemmhofdurchmessern [12].

Dieses Verfahren ist nicht exakt trennscharf, sodass die Agardiffusion lediglich qualitative Aussagen erlaubt und keine MHK-Werte liefert. Für den klinischen Alltag ist dies in der Regel jedoch ausreichend. Auch hier müssen standardisierte Testparameter strikt eingehalten werden, um konsistente und vergleichbare Ergebnisse zu gewährleisten. Durch die Kombination einzelner Antibiotika mit verschiedenen Inhibitoren lassen sich häufig bereits orientierende Aussagen zu Resistenzmechanismen treffen, etwa zu Extended-spectrum-Beta-Laktamasen (ESBL) oder Carbapenemasen.^2^

Die Agardiffusion bietet mehrere Vorteile: geringe apparative Anforderungen, kostengünstige Durchführung, die Möglichkeit, Mischkulturen zu erkennen, sowie validierte Protokolle für eine direkte AST aus positiven Blutkulturen (BK) mit Ablesung nach 4–8 h. Dieses Verfahren wird unten genauer beschrieben (siehe Abschnitt zu schnellen phänotypischen AST-Methoden).

Nachteile sind eine längere Bearbeitungszeit und die eingeschränkte Testbarkeit bestimmter Antibiotika (z. B. Vancomycin bei Staphylokokken), da die Zuordnung des Hemmhofdurchmessers zur MHK nicht zuverlässig ist. Für die bislang aufwendige manuelle Ablesung stehen mittlerweile automatisierte Systeme zur Verfügung, die zudem eine Archivierung der Ergebnisse ermöglichen [13].

### Gradientendiffusionstestung

Die Gradientendiffusion erlaubt die genaue Bestimmung der MHK, basiert jedoch auf den Prinzipien der Agardiffusion [14]. Basis der Gradientendiffusionstestung sind Papier- oder Plastikstreifen, welche mit einem Antibiotikakonzentrationsgradienten beschichtet sind. Diese werden auf ein mit dem zu testenden Isolat beimpftes Medium aufgelegt. Die Höhe des Schnittpunktes eines entstehenden (ellipsoiden) Hemmhofes mit dem Streifen zeigt die MHK des Erregers gegenüber dem getesteten Antibiotikum an. Für viele Antibiotika wurde eine gute Korrelation mit dem Referenzverfahren (Bouillondilution) festgestellt.

Viele Vorteile der Agardiffusion, wie zum Beispiel die gute Erkennbarkeit von Mischkulturen, treffen auch auf die Gradientendiffusionstestung zu. Die höheren Kosten und die aufwendigere Erstellung des Tests erlauben Routinelaboren die Verwendung nur in Einzelfällen. Dennoch ist die Gradientendiffusion das am häufigsten verwendete Verfahren zur MHK-Bestimmung im klinischen Labor.

### Automatisierte Verfahren

Automatisierte AST-Verfahren sind aufgrund der einfachen Handhabung und integrierter softwarebasierter Expertensysteme, welche die detektierten Phänotypen interpretieren, die am häufigsten verwendeten Systeme. Die kommerziell erhältlichen Systeme folgen in miniaturisierter Form dem Prinzip der Bouillondilution [15]. Allerdings werden in den Systemen meist keine vollständigen Verdünnungsreihen getestet, sondern nur Konzentrationen, welche die Kategorisierung in S/I/R erlauben, also Breakpoint-MHK. Nachteilig können die fehlende Flexibilität der Testeinheiten mit den vom Hersteller vorgegebenen Antibiotikazusammenstellungen sein sowie längere Umstellungsphasen bei sich verändernden Interpretationsnormen.

### Schlussfolgerungen zu phänotypischen Resistenztestungen

Während die MHK-bestimmenden Verfahren aufgrund des höheren Arbeitsaufwandes spezielleren Fragestellungen vorbehalten bleiben (wie etwa AST von Pilzen, speziellen Erregern wie Meningokokken oder der Bestätigung außergewöhnlicher Phänotypen), entfällt der größte Teil der Testungen im Routinelabor auf die kategorialen Systeme der automatisierten Verfahren und die Agardiffusion.

Ein großes Problem aller phänotypischen Verfahren bleibt die lange Dauer (16–20 h), bis das Ergebnis vorliegt. Initiale Therapieentscheidungen werden vermutlich auch in absehbarer Zukunft weiterhin empirisch erfolgen müssen. Damit die im Verlauf eintreffenden Ergebnisse der AST zu einer Anpassung der antibiotischen Therapie führen, muss eine regelmäßige entsprechende Reevaluation einer empirischen Therapie Teil antiinfektiver Behandlungen sein.

## Schnelle phänotypische AST-Methoden

Die konventionelle AST erfolgt nach Übernachtinkubation ausgehend von Reinkulturen und führt damit zu einer zeitlichen Verzögerung (Ergebnisse frühestens nach 48 h), die zu einer verlängerten Phase inadäquater antimikrobieller Therapie beitragen kann. In den vergangenen Jahren wurden mehrere Herangehensweisen zur schnellen – „rapid“ – AST (RAST) entwickelt, bei denen die Testung direkt aus der positiven BK erfolgt, ohne den Umweg einer Subkultur auf Festagarmedien. Hierdurch ist es möglich, die Zeit bis zum Vorliegen der Ergebnisse um bis zu 24 h zu verkürzen.

Folgend werden exemplarisch einige der neueren Methoden beschrieben: EUCAST RAST, QuickMIC (Gradientech, Uppsala, Schweden) und Vitek Reveal (bioMérieux, Marcy l’Étoile, Frankreich). Diese Verfahren liefern erste Ergebnisse innerhalb von etwa 4–8 h. Dadurch kann bei resistenten Spezies die Antibiotikatherapie schneller optimiert werden und teilweise konnte bereits ein positiver Trend im Überleben der Patienten gezeigt werden [16–19].

### Agardiffusion-basierte beschleunigte AST – RAST

Die von dem EUCAST entwickelte RAST-Methode basiert technisch auf dem Agardiffusionsverfahren (s. oben). Ausgangspunkt hier ist jedoch nicht ein Erreger von einer Festkultur, sondern Material aus einer positiven Blutkulturflasche. Dieses Material wird auf einem festen Agarmedium ausgestrichen, auf Antibiotikatestplättchen aufgebracht und resultierende Hemmhofdurchmesser werden zu frühen Zeitpunkten (nach 4 h, 6 h, 8 h oder 16–20 h) abgelesen. Die Auswertung erfolgt anhand eines speziell für die Interpretation der Hemmhofdurchmesser zu diesen Zeitpunkten validierten Regelwerks des EUCAST. In mehreren Publikationen konnte gezeigt werden, dass RAST zuverlässige Resultate liefert, im Vergleich zum Goldstandard (Bouillondilution) beziehungsweise zu anderen zum Beispiel semiautomatischen AST-Verfahren in der Mikrobiologie [16, 20–22].

Die Vorteile dieser Herangehensweise liegen in der Einfachheit der Methodik und den geringen Kosten. Da in jedem mikrobiologischen Labor auf bestehende Infrastruktur des Agardiffusionsverfahrens zurückgegriffen werden kann, eignet sich RAST besonders für kleinere oder ressourcenbegrenzte Laboratorien. Gleichzeitig liefert es in vielen Fällen bereits am Tag der BK-Positivität klinisch nutzbare Informationen. Außerdem ist die Methodik vom EUCAST validiert und wird regelmäßig weiterentwickelt (zum Beispiel Validierung neuer Spezies und Substanzen).

Nachteile ergeben sich jedoch aus der bislang eingeschränkten Anzahl validierter Spezies. Zudem können die Hemmhöfe, wenn sie nah am Breakpoint liegen, zu falschen Interpretationen führen. Zusätzlich können sie aufgrund der kurzen Inkubationszeit nur schwer ablesbar sein oder auch in der „area of technical uncertainty“ (ATU) liegen, sodass die Substanzen nicht sicher interpretiert werden können. Insbesondere für Piperacillin/Tazobactam sind hohe ATU-Raten beschrieben [16, 20]. Auch sind sehr genaue manuelle Ablesungen notwendig und eine vollständige Automatisierbarkeit ist bislang nicht gegeben, was die Skalierbarkeit einschränkt.

### Mikroskopie-basierte AST – QuickMIC

Das QuickMIC-System von Gradientech basiert auf mikroskopischer Analyse des bakteriellen Wachstums in Agarose mit einem linearen Antibiotikagradienten. Dafür wird Material aus einer positiven Blutkulturflasche in eine Testkassette überführt, welche in ein spezielles Assaysystem eingebracht wird. Hier wird die Empfindlichkeit gegenüber 12 unterschiedlichen Antibiotika durch kontinuierliche Analyse des bakteriellen Wachstums mittels Mikroskopie evaluiert. Dadurch kann innerhalb weniger Stunden (etwa 3–4 h) eine MHK ermittelt werden. QuickMIC ist schneller als die EUCAST-RAST-Methode. Zudem erfolgen die Ablesung und Dateninterpretation in einer automatisierten Form. Da das Verfahren mehr Spezies als EUCAST RAST abgedeckt, wird der klinische Anwendungsbereich deutlich erweitert.

In den ersten Studien konnte eine gute Übereinstimmung der QuickMIC-Ergebnisse mit den Referenz-AST gezeigt werden. Bei insgesamt noch begrenzten Erfahrungen im klinischen Alltag deutet aktuelle Evidenz auf Probleme hin, vor allem bei der Prüfung von Piperacillin/Tazobactam und Meropenem [23, 23, 24, 24, 25].

### Andere RAST-Verfahren – Vitek Reveal

Im Vergleich zu den bisher vorgestellten RAST-Systemen macht sich Vitek Reveal von bioMérieux die Detektion von volatilen metabolischen Produkten (VMB), die Bakterien bei Wachstum absondern, zunutze. Das System nutzt dafür ein Array von Sensoren, welche bei der Erkennung des Ziel-VMB mit einer quantitativen Farbänderung reagieren und so die Erfassung des mikrobiellen Wachstums in Echtzeit ermöglichen. Die Zeit bis zum Ergebnis liegt bei ca. 5,5–6 h. Eine aktuelle Studie zeigte gute MHK-Übereinstimmung mit der Bouillondilution und eine ähnliche kategorische Übereinstimmungsrate wie RAST (98,3 % kategorische Übereinstimmung bei Vitek Reveal beziehungsweise 98,2 % bei RAST; [26]). Dies trifft auch auf die Prüfung von Piperacillin/Tazobactam zu [26].

### Schlussfolgerungen zu RAST-Verfahren

Die genannten Methoden verdeutlichen, dass es mittlerweile eine Reihe unterschiedlichster Ansätze gibt, die die Zeitspanne zwischen BK-Positivität und vorliegenden AST-Ergebnissen erheblich verkürzen können. EUCAST RAST bietet hier einen kostengünstigen, breit implementierbaren Ansatz mit guter Leistung. Systeme wie QuickMIC und Vitek Reveal bieten den Vorteil, dass sie automatisiert sind, quantitative, schnelle Ergebnisse liefern und diese automatisch interpretieren, allerdings mit dem Nachteil hoher Investitions- und Betriebskosten. Außerdem sind alle genannten Herangehensweisen auf bestimmte validierte Spezies beziehungsweise Antibiotika beschränkt.

Allen Verfahren ist zudem gemeinsam, dass ihr klinischer Nutzen bisher noch nicht klar nachgewiesen wurde. Auch fehlen gesundheitsökonomische Berechnungen, die besonders für die Systeme mit hohen Investitionskosten von großer Wichtigkeit sein werden. In diesem Zusammenhang spielt das sogenannte Diagnostic Stewardship eine bedeutende Rolle, um möglichst individualisiert die Patienten zu identifizieren, die am meisten von einem neuen (gegebenenfalls teuren) Test profitieren könnten.

Besonders hervorzuheben ist, dass der Erfolg der schnellen Verfahren von der Einbettung in ein funktionierendes ABS-Programm abhängt. Nur wenn die Ergebnisse zügig kommuniziert werden und in therapeutische Entscheidungen einfließen, kann das Potenzial dieser Technologien vollkommen ausgeschöpft werden. Angesichts zunehmender Resistenzen und der Notwendigkeit einer präzisen, individualisierten Antibiotikatherapie werden diese Verfahren in den kommenden Jahren eine immer wichtigere Rolle in der mikrobiologischen Routinediagnostik einnehmen.

## Vorhersage der Antibiotikaempfindlichkeit mittels molekularer Nachweisverfahren

Ein signifikanter Nachteil phänotypischer AST-Verfahren ist die methodeninhärente Langsamkeit. Dies führt zu einer Verzögerung des diagnostischen Prozesses und es resultiert die Gefahr, dass nicht wirksame empirische Antibiotika unnötig lange verabreicht werden. Der direkte Nachweis von Resistenzmarkern mittels molekularer Verfahren und die hieraus abgeleitete Vorhersage einer phänotypischen Empfindlichkeit können diese Limitierungen phänotypischer Verfahren überwinden.

### Methoden zum Nachweis resistenzassoziierter Marker

Molekulare Verfahren zum Nachweis resistenzassoziierter Marker beruhen überwiegend auf der Detektion spezifischer Nukleinsäuresequenzen. Die Polymerase-Kettenreaktion (PCR) bildet dabei den methodischen Standard, da sie durch Amplifikation definierter Resistenzgene eine hochspezifische Identifikation und Vorhersage einer Resistenz ermöglicht (zum Beispiel *mecA* zur Vorhersage einer Methicillinresistenz [27]). Isothermale Amplifikationstechniken wie die Loop-mediated Isothermal Amplification (LAMP) arbeiten bei konstanter Temperatur und reduzieren dadurch den apparativen Aufwand (zum Beispiel eazyplex SuperBug CRE Test [28]). Die Verfahren zeichnen sich durch eine kurze Dauer bis zum Ergebnis und hohe analytische Empfindlichkeit aus, sind jedoch anfällig für unspezifische Amplifikationen [29].

Die Ganzgenomsequenzierung von Pathogenen (WGS) mittels Methoden der Hochdurchsatzsequenzierung (Next Generation Sequencing, NGS) erlaubt eine umfassende, genomweite Analyse genetischer Resistenzdeterminanten [30]. Hierbei geht die Informationstiefe deutlich über die der PCR hinaus, da nicht nur die bloße An- oder Abwesenheit genetischer Marker analysiert werden kann, sondern auch resistenzassoziierte Mutationen auf Ebene einzelner Nukleotide dargestellt werden können. WGS bietet vor diesem Hintergrund unmittelbare Vorteile gegenüber den Amplifikations-basierten Technologien [31]. Aufgrund der hohen Kosten und der langen Umsatzzeiten ist die WGS-basierte Analytik aktuell nicht auf breiter Basis in die Routineanalytik integriert. Mit abnehmenden Kosten, der Weiterentwicklung von Verfahren und Verfügbarkeit robuster bioinformatischer Analysemethoden ist zu erwarten, dass die NGS-basierte Erregeranalytik weiter in den Alltag der Routineanalytik einziehen wird [32].

### Klinische Nutzbarkeit bei grampositiven Erregern

Klinisch bedeutsame grampositive Erreger mit erworbenen Resistenzen sind Methicillin-resistente *Staphylococcus aureus* (MRSA) und Vancomycin-resistente Enterokokken (VRE). Die genetische Basis der Methicillin- und Glykopeptidresistenz bei Staphylokokken beziehungsweise Enterokokken ist gut erforscht. Nur eine begrenzte Zahl genetischer Marker ist für die Expression eines entsprechenden Resistenzphänotyps relevant (Methicillinresistenz: *mecA* und *mecC*; Glykopeptidresistenz: *vanA* und *vanB*). Der Nachweis dieser Gene lässt die Vorhersage eines entsprechenden Phänotyps sicher zu, während ihr Fehlen mit hoher Präzision eine entsprechende Resistenz ausschließt. Hieraus lassen sich direkt klinisch nutzbare Informationen ableiten (Nachweis *mecAC*: Notwendigkeit zum Beispiel einer Glykopeptidtherapie; Ausschluss *mecAC*: optimierte Beta-Laktam-Antibiotikatherapie; [33]). Aus diesem Grund ist der molekulare Nachweis von *mecAC* und *vanAB* wichtiger Bestandteil von Verfahren zur Aufarbeitung positiver BK [34, 35].

Vollintegrierte Systeme (zum Beispiel GenXpert, Biofire Filmarray, Verigene, GenMark Dx ePlex) stehen zur Verfügung, die zum Teil zusätzlich zum Nachweis von *mecAC* und *vanAB* auch eine Speziesidentifikation ermöglichen [27]. Ihr Einsatz in der Sepsisdiagnostik kann die Zeit bis zur Einleitung einer optimalen antimikrobiellen Therapie um mehr als 24 h verkürzen und dadurch die diagnostische Effizienz sowie die Versorgungsqualität erheblich verbessern [36].

Einige der genannten Systeme werden zudem genutzt, um Resistenzmarker kulturunabhängig, also direkt aus klinischem Material, zu detektieren. Dabei sind die genetischen Marker entweder Bestandteil syndromischer PCR-Panels [37] oder dienen dem isolierten Nachweis spezifischer Zielstrukturen im Rahmen krankenhaushygienischer Überwachungen [38]. In diesen Anwendungskontexten, insbesondere bei potenziell polymikrobiellen Infektionen oder komplexen mikrobiellen Gemeinschaften, ist eine eindeutige Zuordnung eines nachgewiesenen Resistenzgens zu einer bestimmten Spezies jedoch nicht immer möglich. Eine weitere Limitierung besteht in der Möglichkeit falsch-positiver Befunde. Aus diesem Grund sollte auch bei Durchführung eines molekularen Resistenzmarkernachweises in jedem Fall eine phänotypische AST angestrebt werden [39].

### Klinische Nutzbarkeit bei gramnegativen Erregern

Die zunehmende Ausbreitung multiresistenter gramnegativer Erreger macht es erforderlich, die Wirksamkeit empirischer Antibiotikatherapien zügig zu überprüfen. Tatsächlich ist es auch bei gramnegativen Erregern wie *Escherichia* (*E.*) *coli, Klebsiella *(*K.*) *pneumoniae* und *Pseudomonas* (*P.*) *aeruginosa* möglich, durch den molekularen Nachweis von Beta-Laktamasen die Wirksamkeit von Cephalosporinen und Carbapenemen vorherzusagen. Analog zu den bei grampositiven Erregern beschriebenen Einsatzgebieten stellen molekulare Testverfahren damit eine Möglichkeit des schnellen und sensitiven Nachweises entsprechender Resistenzmarker im Rahmen klinisch-mikrobiologischer wie auch krankenhaushygienischer Analytik.

Tatsächlich sind kommerzielle Testsysteme verfügbar, die den Nachweis der auch in Deutschland häufigsten ESBL wie CTX‑M und Carbapenemasen (zum Beispiel *K.-pneumoniae*-Carbapenemasen (KPC), Oxa-48, NDM, VIM) erlauben. Diese Testsysteme, die auf Amplifikationsreaktion oder auf immunologischen Testprinzipien basieren, können für die Differenzierung gramnegativer Erreger aus positiven BK eingesetzt werden. Klinische Studien zeigen, dass der beschleunigte Nachweis einer KPC-vermittelten Carbapenemresistenz bei einer *K.-pneumoniae*-Sepsis die Dauer bis zum Einsatz eines geeigneten Antibiotikums signifikant verkürzt [18, 40–42] und die Mortalität signifikant senkt [43].

Eine wesentliche Herausforderung bei der molekularen Vorhersage gramnegativer Resistenz besteht aufgrund der Heterogenität der Mechanismen, die zum Beispiel in einem Cephalosporin- oder Carbapenem-resistenten Phänotyp münden: Es ist aktuell sehr schwer bis unmöglich, alleine basierend auf dem Nachweis eines bestimmten Markers oder dessen Fehlen mit ausreichender Sicherheit einen resistenten Phänotyp vorherzusagen oder auszuschließen [42]. Dies limitiert bei gramnegativen Infektionserregern die Nutzbarkeit von Verfahren, die durch den Nachweis einzelner Gene resistente Phänotypen vorhersagen. WGS könnte es zukünftig möglich machen, die Beschränkung der Analytik aufzulösen, die auf dem Nachweis einer begrenzten Anzahl von einzelnen Markern basiert [44]. Hierbei muss aber beachtet werden, dass die Ausprägung eines resistenten Phänotyps auch durch die dynamische Expression einzelner Gene modifiziert werden kann [45]. Dies könnte die alleinige Nutzung genomischer Informationen signifikant limitieren.

### Schlussfolgerungen zur Vorhersage der Antibiotikaempfindlichkeit mittels molekularer Nachweisverfahren

Verfahren zum Nachweis von Resistenzmarkern sind von zentraler Bedeutung im klinisch-mikrobiologischen Labor. Sie verbessern die Analyse positiver BK und ermöglichen den kulturunabhängigen Nachweis von Resistenzdeterminanten. Dieser Aspekt ist insbesondere im Rahmen von krankenhaushygienischen Screening-Untersuchungen relevant.

Molekulare Methoden eignen sich gut zur sicheren Identifikation resistenter grampositiver Erreger (MRSA, VRE). Aufgrund komplexer Mechanismen gramnegativer Resistenz sind Marker-basierte Assays hier bislang wenig zuverlässig; phänotypische AST bleiben erforderlich. Zukünftig könnten WGS-Ansätze an Bedeutung gewinnen [46].

## KI in der Resistenztestung: Mögliche Einsatzoptionen

Antibiotikaresistenz ist eine zentrale Herausforderung der modernen Medizin. Klassische phänotypische Verfahren gelten weiterhin als technischer Goldstandard, stoßen aber hinsichtlich Geschwindigkeit, Standardisierung und Skalierbarkeit an ihre Grenzen. Parallel eröffnen molekulargenetische Methoden – insbesondere WGS und „microbial cell free DNA“ (mcfDNA) – neue Möglichkeiten, Resistenzmechanismen auf molekularer Ebene zu erkennen.

In diesem Spannungsfeld spielt KI eine immer wichtigere Rolle, da sie aus komplexen, multidimensionalen Datensätzen Muster erkennen und Resistenzvorhersagen automatisiert treffen kann. Eine aktuelle Übersicht hebt hervor, dass KI-gestützte Analyse von WGS-Daten der Schlüssel ist, um Resistenzanalysen aus der Forschungsumgebung in die klinische Routine zu überführen. Auch können hiermit gegebenenfalls die Entstehung neuer Resistenzen, deren Übertragung sowie Verbreitung in einem „One-Health“-Umfeld (Verbindung der Gesundheit von Menschen, Tieren, Pflanzen und deren Umwelt) besser verstanden werden. Damit wird deutlich, dass der Einsatz von KI in der Resistenzdiagnostik nicht nur ein technisches, sondern ein translationales Thema ist [47].

### KI in der genotypischen Resistenzanalyse

Die genotypische Resistenzdetektion stützt sich primär auf das Erkennen bekannter Resistenzgene oder Mutationen (zum Beispiel *bla*_*CTX‑M*_, *mecAC, vanAB*, Porin- oder Effluxmutationen). Traditionelle Werkzeuge wie AMRFinderPlus oder ResFinder liefern regelbasierte Interpretationen, stoßen aber bei komplexen oder neuartigen Mechanismen an ihre Grenzen [32, 48, 49]. Hier könnte KI einen wichtigen Beitrag leisten. Basierend auf genomweiten Assoziationsstudien könnten bisher unbekannte Determinanten resistenter Phänotypen identifiziert werden, wie zum Beispiel regulatorische Phänomene oder spezifische Kombinationen von definierten Single Nucleotide Polymorphisms (SNP; [50–52]). Ein weiteres Beispiel ist das Tool PorinPredict [53]. Das Modell sagt die Meropenemempfindlichkeit von *P. aeruginosa* anhand von WGS-Daten vorher, indem der Verlust oder die Mutation des Porins OprD analysiert wird.

Diese genannten Arbeiten zeigen, dass die KI-gestützte Detektion von Resistenzmechanismen weit über die einfache Aussage zur An- oder Abwesenheit bestimmter Marker hinausgehen kann.

### KI in der phänotypischen Resistenzanalyse

Trotz des Fortschritts in der Genomik bleibt die phänotypische AST der diagnostische Referenzstandard. KI bietet hier vielfältige Anwendungsmöglichkeiten: von der automatisierten Zonenmessung über Schnelltest-Interpretation bis hin zur Auswertung spektroskopischer Signaturen.

#### Automatisierte Interpretation von phänotypischen Tests

Grundlage für einen effektiven Einsatz von KI in der kulturbasierten Mikrobiologie ist eine Digitalisierung des Arbeitsablaufs. Hier hat bereits die Laborautomatisation, ohne Einsatz von KI, gezeigt, dass viele bisher manuelle Prozesse übernommen werden können, wie zum Beispiel die Inspektion von Agarmedien zum Nachweis von Wachstum, Einschätzung der Koloniezahlen und davon abhängige Entscheidungen zur diagnostischen Weiterverarbeitung [54–56]. Solche Systeme reduzieren die Variabilität zwischen Laborpersonal deutlich und verkürzen die Auswertungszeit [54].

Bei der phänotypischen Resistenzanalyse zeigen erste Studien, dass das Training des mittlerweile weltbekannten Large Language Model ChatGPT mithilfe von Agardiffusionstesten nachfolgend bei der Interpretation von Antibiogrammen unterstützen kann [57]. Der so geschulte „EUCAST-GPT-Expert“ konnte anhand von Bildern von Agardiffusionstesten zugrunde liegende Resistenzen, wie ESBL oder Carbapenemasen, zuverlässig identifizieren (Sensitivität: 95,4 % bzw. 100 %), allerdings war die Spezifität deutlich schlechter (69,2 % bzw. 98,8 %; [57]).

#### MALDI-TOF-basierte Resistenzvorhersage

Ein weiterer innovativer Einsatzbereich ist die Kombination von MALDI-TOF-Massenspektrometrie mit KI-gestützter Mustererkennung. In einer Studie wurde ein Modell mit 300.000 massenspektrometrischen Daten und entsprechenden phänotypischen Empfindlichkeitsdaten trainiert und anhand von typischen Pathogenen (u. a. *S. aureus, E. coli* und *K. pneumoniae*) validiert [58]. Für *S. aureus *konnte hier eine gute Vorhersage der Oxacillin-Empfindlichkeit erzielt werden. Ähnlich gut ist, laut der Publikation, die Vorhersage der Ceftriaxon-Resistenz bei *E. coli* und *K. pneumoniae*. Andere Veröffentlichungen zeigen eine Anwendbarkeit der Kombination von Massenspektrometrie und KI auch in Bezug auf *P. aeruginosa* [59] und Carbapenemase-Detektion bei *K. pneumoniae *[60]. In Zukunft erlauben solche Herangehensweisen eventuell die Zeit bis zu einer, zumindest grob orientierenden Resistenzvorhersage deutlich zu verkürzen.

### Anwendungsszenario

In einem mikrobiologischen Labor könnten im Rahmen der Laborautomatisation bereits durch regelmäßige Inspektion der Platten – auch außerhalb der Kernarbeitszeit – Inkubationszeiten verkürzt und die Analyse weniger variabel gemacht werden. In dem Zusammenhang könnten zum Beispiel genotypische, massenspektrometrische oder phänotypische Daten, die in digitalisierter Form vorliegen, von einer KI verarbeitet werden und gegebenenfalls weiterführende Tests oder Aussagen zu möglicher Resistenz und Übertragungen innerhalb des Krankenhauses getroffen werden. Am Ende dieses deutlich schnelleren Arbeitsablaufs steht die Kommunikation an behandelnde Ärzte, die dann eine Therapieoptimierung durchführen können.

### Herausforderungen

Die Aussagekraft der KI-Systeme hängt wesentlich von der Datenqualität ab. Ohne korrekt kalibrierte Referenzwerte sind KI-Modelle anfällig für Fehlklassifikationen [47]. Zusätzlich stellt sich auch die Frage der Generalisierbarkeit. Bisherige Publikationen haben die Modelle mit ihren lokalen Daten trainiert und getestet. Aussagen dazu, wie diese Modelle in einem anderen nationalen Labor oder, noch kritischer, im internationalen Vergleich abschneiden würden, gibt es bisher nicht.

Zusätzlich zeigen sich bereits Schwächen der Modelle. Der oben vorgestellte EUCAST-GPT-Expert hatte zwar eine gute Sensitivität, aber eine schlechte Spezifität, was zu Nachtestungen, Unsicherheiten und einer verspäteten finalen AST führen könnte [57]. Zudem zeigt sich weltweit ein deutliches Ungleichgewicht: Komplexe Datensätze wie NGS- oder Massenspektrometrie-Daten stammen überwiegend aus Ländern mit hohem Einkommen. In Ländern mit niedrigem und mittlerem Einkommen sind solche Daten hingegen stark unterrepräsentiert. Gerade die Länder, die unter einer besonders hohen Antibiotikaresistenzlast leiden, sind daher auf längere Sicht von den möglichen Vorteilen des Einsatzes von KI in der klinischen Mikrobiologie abgeschnitten [3].

Die meisten Labore sind derzeit noch nicht automatisiert und viele Analysesysteme können nicht miteinander kommunizieren. Eine vollständige Integration in KI-gestützte Arbeitsabläufe ist daher im Routinebetrieb bislang nicht realisierbar. Auch die erheblichen regulatorischen Anforderungen (etwa durch die Verordnung über In-vitro-Diagnostika (IVDR) oder die Prüfung durch die Food and Drug Administration der USA (FDA)) wurden bisher kaum adressiert. Zudem konnte selbst für bestehende RAST-Ansätze weder ein eindeutiger Überlebensvorteil für Patienten noch ein klarer gesundheitsökonomischer Nutzen belegt werden. Obwohl der Einsatz von KI in der medizinischen Mikrobiologie zweifellos großes Potenzial besitzt, ist der Weg bis zur breiten Routineanwendung noch weit.

## Fazit

Die Empfindlichkeitsbewertung klinischer Isolate ist ein entscheidender Baustein für das Management von Infektionen und Grundlage einer rationalen Antibiotikatherapie entlang der Prinzipien des Antimicrobial Stewardship (ABS). Zudem ist der Nachweis spezifischer Resistenzphänotypen essenzielle Grundlage einer effektiven Infektionsprävention im Krankenhaus. In den vergangenen Jahren haben sich parallel zu den klinischen Routinemethoden neue Ansätze zur Bewertung der Antibiotikaempfindlichkeit entwickelt.

Methoden der beschleunigten Empfindlichkeitstestung (AST) und der Vorhersage spezifischer Resistenzphänotypen mittels molekularer Assays zur Detektion von genetischen Resistenzdeterminanten machen es möglich, innerhalb weniger Stunden klinisch nutzbare Erkenntnisse zu liefern. Damit kann die Zeit bis zum Ergebnis um bis zu 48 h oder sogar mehr verkürzt werden. Dies ist insbesondere bei lebensbedrohlichen Infektionen wie einer Sepsis eine signifikante Verbesserung und es besteht kein Zweifel, dass beschleunigte Verfahren ein essenzieller Baustein der Patientenversorgung sein müssen.

Allerdings sind neue Verfahren mit zum Teil hohen Kosten verbunden. Zudem werden erhebliche Anpassungen der Routineprozesse eines Labors notwendig. Daher bleiben traditionelle AST-Methoden die Basis der Analytik im klinisch-mikrobiologischen Labor. Methoden wie schnelle AST und molekulare Tests sollen gezielt nur dann eingesetzt werden, wenn ein entscheidender klinischer Vorteil zu erwarten ist. Zudem ist wichtig, dass die Erweiterung der AST-Verfahren innerhalb des Labors zwingend verbunden sein muss mit einer Überprüfung der präanalytischen (zum Beispiel Indikationsstellung, Probentransportdauer, Laboröffnungszeiten) und postanalytischen Prozesse (zum Beispiel Befundkommunikation, Übersetzung neuer Informationen in angemessene klinische Maßnahmen). Zum Teil erscheint es möglich, dass hierbei elektronische Unterstützungssysteme auch unter Einsatz von KI eine wichtige Rolle spielen könnten.

## Footnotes

1 https://www.eucast.org/fileadmin/eucast/pdf/disk_test_documents/2025/Manual_v_13.0_EUCAST_Disk_Test_2025.pdf, zuletzt aufgerufen am 18.11.2025.

2 https://www.eucast.org/bacteria/important-additional-information/resistance-detection/; zuletzt aufgerufen am 18.11.2025.

## References

[1] Marchaim D, Gottesman T, Schwartz O et al (2010) National multicenter study of predictors and outcomes of bacteremia upon hospital admission caused by Enterobacteriaceae producing extended-spectrum beta-lactamases. Antimicrob Agents Chemother 54:5099–5104. 10.1128/AAC.00565-10 20837757 10.1128/AAC.00565-10 PMC2981239

[2] Kang CI, Kim SH, Park WB et al (2005) Bloodstream infections caused by antibiotic-resistant gram-negative bacilli: risk factors for mortality and impact of inappropriate initial antimicrobial therapy on outcome. Antimicrob Agents Chemother 49:760–766. 10.1128/AAC.49.2.760-766.2005 15673761 10.1128/AAC.49.2.760-766.2005 PMC547233

[3] Collaborators GBDAR (2024) Global burden of bacterial antimicrobial resistance 1990-2021: a systematic analysis with forecasts to 2050. Lancet 404:1199–1226. https://doi.org/10.1016/S0140–6736(24)01867‑1 39299261 10.1016/S0140-6736(24)01867-1 PMC11718157

[4] Van Heuverswyn J, Valik JK, van der Werff S D, Hedberg P, Giske C, Naucler P (2023) Association Between Time to Appropriate Antimicrobial Treatment and 30-day Mortality in Patients With Bloodstream Infections: A Retrospective Cohort Study. Clin Infect Dis 76:469–478. 10.1093/cid/ciac727 36065752 10.1093/cid/ciac727 PMC9907509

[5] Andrews JM (2001) Determination of minimum inhibitory concentrations. J Antimicrob Chemother 48(Suppl 1):5–16. 10.1093/jac/48.suppl_1.5 11420333 10.1093/jac/48.suppl_1.5

[6] Kahlmeter G, Turnidge J (2023) Wild-type distributions of minimum inhibitory concentrations and epidemiological cut-off values-laboratory and clinical utility. Clin Microbiol Rev 36:e0010022. 10.1128/cmr.00100-22 38038445 10.1128/cmr.00100-22 PMC10732016

[7] Hombach M, Ochoa C, Maurer FP, Pfiffner T, Bottger EC, Furrer R (2016) Relative contribution of biological variation and technical variables to zone diameter variations of disc diffusion susceptibility testing. J Antimicrob Chemother 71:141–151. 10.1093/jac/dkv309 26462987 10.1093/jac/dkv309

[8] van Belkum A, Bachmann TT, Ludke G et al (2019) Developmental roadmap for antimicrobial susceptibility testing systems. Nat Rev Microbiol 17:51–62. 10.1038/s41579-018-0098-9 30333569 10.1038/s41579-018-0098-9 PMC7138758

[9] Fleming A (2001) On the antibacterial action of cultures of a penicillium, with special reference to their use in the isolation of B. influenzae. 1929. Bull World Health Organ 79:780–790 11545337 PMC2566493

[10] Wiegand I, Hilpert K, Hancock RE (2008) Agar and broth dilution methods to determine the minimal inhibitory concentration (MIC) of antimicrobial substances. Nat Protoc 3:163–175. 10.1038/nprot.2007.521 18274517 10.1038/nprot.2007.521

[11] Hackel MA, Tsuji M, Yamano Y, Echols R, Karlowsky JA, Sahm DF (2019) Reproducibility of broth microdilution MICs for the novel siderophore cephalosporin, cefiderocol, determined using iron-depleted cation-adjusted Mueller-Hinton broth. Diagn Microbiol Infect Dis 94:321–325. 10.1016/j.diagmicrobio.2019.03.003 31029489 10.1016/j.diagmicrobio.2019.03.003

[12] Turnidge J, Paterson DL (2007) Setting and revising antibacterial susceptibility breakpoints. Clin Microbiol Rev 20:391–408. 10.1128/CMR.00047-06 17630331 10.1128/CMR.00047-06 PMC1932754

[13] Callebaut K, Stoefs A, Emmerechts K et al (2024) Evaluation of Automated Disk Diffusion Antimicrobial Susceptibility Testing Using Radian(R) In-Line Carousel. Curr Microbiol 81:196. 10.1007/s00284-024-03710-z 38816509 10.1007/s00284-024-03710-z PMC11139706

[14] Salam MA, Al-Amin MY, Pawar JS, Akhter N, Lucy IB (2023) Conventional methods and future trends in antimicrobial susceptibility testing. Saudi J Biol Sci 30:103582. 10.1016/j.sjbs.2023.103582 36852413 10.1016/j.sjbs.2023.103582 PMC9958398

[15] Gajic I, Kabic J, Kekic D et al (2022) Antimicrobial Susceptibility Testing: A Comprehensive Review of Currently Used Methods. Antibiotics (Basel). 10.3390/antibiotics11040427 35453179 10.3390/antibiotics11040427 PMC9024665

[16] Berinson B, Olearo F, Both A et al (2021) EUCAST rapid antimicrobial susceptibility testing (RAST): analytical performance and impact on patient management. J Antimicrob Chemother. 10.1093/jac/dkab02610.1093/jac/dkab026 33585908 10.1093/jac/dkab026

[17] Banerjee R, Humphries R (2021) Rapid Antimicrobial Susceptibility Testing Methods for Blood Cultures and Their Clinical Impact. Front Med (Lausanne) 8:635831. 10.3389/fmed.2021.635831 33777978 10.3389/fmed.2021.635831 PMC7987685

[18] Banerjee R, Giri A, Komarow L et al (2025) Impact of rapid antibiotic susceptibility testing for Gram-negative bacteremia varies by pathogen type and resistance: a secondary analysis of the RAPIDS GN trial. Microbiol Spectr 13:e0178924. 10.1128/spectrum.01789-24 39679788 10.1128/spectrum.01789-24 PMC11792449

[19] Del Corpo O, Senecal J, Hsu JM, Lawandi A, Lee TC (2023) Rapid phenotypic testing for detection of carbapenemase- or extended-spectrum ss-lactamase-producing Enterobacterales directly from blood cultures: a systematic review and meta-analysis. Clin Microbiol Infect 29:1516–1527. 10.1016/j.cmi.2023.09.007 37722531 10.1016/j.cmi.2023.09.007

[20] Akerlund A, Jonasson E, Matuschek E et al (2020) EUCAST rapid antimicrobial susceptibility testing (RAST) in blood cultures: validation in 55 European laboratories. J Antimicrob Chemother 75:3230–3238. 10.1093/jac/dkaa333 32789506 10.1093/jac/dkaa333 PMC7566356

[21] Jonasson E, Matuschek E, Kahlmeter G (2020) The EUCAST rapid disc diffusion method for antimicrobial susceptibility testing directly from positive blood culture bottles. J Antimicrob Chemother 75:968–978. 10.1093/jac/dkz548 32016342 10.1093/jac/dkz548 PMC7069491

[22] Cherkaoui A, Schorderet D, Azam N et al (2022) Fully Automated EUCAST Rapid Antimicrobial Susceptibility Testing (RAST) from Positive Blood Cultures: Diagnostic Accuracy and Implementation. J Clin Microbiol 60:e0089822. 10.1128/jcm.00898-22 36173195 10.1128/jcm.00898-22 PMC9580353

[23] Morecchiato F, Chilleri C, Antonelli A, Malmberg C, Giani T, Rossolini GM (2025) Evaluation of QuickMIC system for rapid antimicrobial susceptibility testing of Gram-negative pathogens from positive blood cultures, including strains producing extended-spectrum beta-lactamases and carbapenemases. Microbiol Spectr 13:e0037025. 10.1128/spectrum.00370-25 40492764 10.1128/spectrum.00370-25 PMC12210996

[24] Berinson B, Davies E, Torpner J et al (2024) A multicenter evaluation of a novel microfluidic rapid AST assay for Gram-negative bloodstream infections. J Clin Microbiol 62:e0045824. 10.1128/jcm.00458-24 39324811 10.1128/jcm.00458-24 PMC11481479

[25] Malmberg C, Torpner J, Fernberg J et al (2022) Evaluation of the Speed, Accuracy and Precision of the QuickMIC Rapid Antibiotic Susceptibility Testing Assay With Gram-Negative Bacteria in a Clinical Setting. Front Cell Infect Microbiol 12:758262. 10.3389/fcimb.2022.758262 35402290 10.3389/fcimb.2022.758262 PMC8984463

[26] Squitieri D, Menchinelli G, Magri C et al (2025) Improving time-to-result: head-to-head comparison of three rapid AST systems for Gram-negative bacteremia, including the newly developed VITEK REVEAL. J Clin Microbiol e0105025. 10.1128/jcm.01050-25 10.1128/jcm.01050-25 PMC12607571 41065398

[27] Mizusawa M, Carroll KC (2023) Recent updates in the development of molecular assays for the rapid identification and susceptibility testing of MRSA. Expert Rev Mol Diagn 23:679–699. 10.1080/14737159.2023.2234823 37419696 10.1080/14737159.2023.2234823

[28] Comini S, Bianco G, Boattini M et al (2022) Evaluation of a diagnostic algorithm for rapid identification of Gram-negative species and detection of extended-spectrum beta-lactamase and carbapenemase directly from blood cultures. J Antimicrob Chemother 77:2632–2641. 10.1093/jac/dkac230 35851808 10.1093/jac/dkac230

[29] Berneking L, Asar L, Both A et al (2021) Performance of a loop-mediated isothermal amplification assay (Isoplex CRE-ART) to detect common carbapenemase-encoding genes in Gram-negative bacteria. J Med Microbiol. 10.1099/jmm.0.001379 34251298 10.1099/jmm.0.001379

[30] Hayden MK, Sansom SE, Snitkin ES (2025) Genome sequencing for prevention of health-care-associated bacterial infections. Nat Rev Microbiol. 10.1038/s41579-025-01254-y10.1038/s41579-025-01254-y 41214237 10.1038/s41579-025-01254-y

[31] Yee R, Simner PJ (2022) Next-Generation Sequencing Approaches to Predicting Antimicrobial Susceptibility Testing Results. Clin Lab Med 42:557–572. 10.1016/j.cll.2022.09.011 36368782 10.1016/j.cll.2022.09.011

[32] Sherry NL, Lee JYH, Giulieri SG et al (2025) Genomics for antimicrobial resistance-progress and future directions. Antimicrob Agents Chemother 69:e0108224. 10.1128/aac.01082-24 40227048 10.1128/aac.01082-24 PMC12057382

[33] Berinson B, Both A, Berneking L et al (2021) Usefulness of Biofire FilmArray BCID2 for blood culture processing in clinical practice. J Clin Microbiol. 10.1128/JCM.00543-2110.1128/JCM.00543-21 33980648 10.1128/JCM.00543-21 PMC8373244

[34] Peri AM, Ling W, Furuya-Kanamori L, Harris PNA, Paterson DL (2022) Performance of BioFire Blood Culture Identification 2 Panel (BCID2) for the detection of bloodstream pathogens and their associated resistance markers: a systematic review and meta-analysis of diagnostic test accuracy studies. BMC Infect Dis 22:794. 10.1186/s12879-022-07772-x 36266641 10.1186/s12879-022-07772-x PMC9585790

[35] Both A, Berneking L, Berinson B et al (2020) Rapid identification of the vanA/vanB resistance determinant in Enterococcus sp. from blood cultures using the Cepheid Xpert vanA/vanB cartridge system. Diagn Microbiol Infect Dis 96:114977. 10.1016/j.diagmicrobio.2019.114977 31954596 10.1016/j.diagmicrobio.2019.114977

[36] Chen K, Malik AA, Sheng YJ et al (2021) Clinical Utility of Molecular Tests for Guiding Therapeutic Decisions in Bloodstream Staphylococcal Infections: A Meta-Analysis. Front Pediatr 9:713447. 10.3389/fped.2021.713447 34422731 10.3389/fped.2021.713447 PMC8374148

[37] Ramanan P, Bryson AL, Binnicker MJ, Pritt BS, Patel R (2018) Syndromic Panel-Based Testing in Clinical Microbiology. Clin Microbiol Rev 3110.1128/CMR.00024-17 10.1128/CMR.00024-17 PMC5740973 29142077

[38] Maurin E, Ranc AG, Abad L et al (2020) Performance of the Hologic Panther Fusion(R) MRSA Assay for the nasal screening of methicillin-sensitive and methicillin-resistant Staphylococcus aureus carriage. Eur J Clin Microbiol Infect Dis 39:2169–2176. 10.1007/s10096-020-03968-8 32643026 10.1007/s10096-020-03968-8

[39] Jacqmin H, Schuermans A, Desmet S, Verhaegen J, Saegeman V (2017) Performance of three generations of Xpert MRSA in routine practice: approaching the aim? Eur J Clin Microbiol Infect Dis 36:1363–1365. 10.1007/s10096-017-2939-2 28321579 10.1007/s10096-017-2939-2

[40] Oudiane L, Benyahia M, Salipante F et al (2025) Clinical impact of the BCID2 and rapid AST VITEK(R) REVEALTM on antibiotic optimisation in critically ill patients with Gram-negative bloodstream infections: a quasi-experimental pre/post interventional study. J Antimicrob Chemother 80:2665–2675. 10.1093/jac/dkaf271 40754712 10.1093/jac/dkaf271

[41] Wolk DM, Parrott JS, Babady NE et al. (2025) The American Society for Microbiology’s evidence-based laboratory medicine practice guidelines for the diagnosis of bloodstream infections using rapid tests: a systematic review and meta-analysis. Clin Microbiol Rev 38:e0013724. https://doi.org/10.1128/cmr.00137-24 10.1128/cmr.00137-24 PMC12424361 40522178

[42] Peri AM, Chatfield MD, Ling W, Furuya-Kanamori L, Harris PNA, Paterson DL (2024) Rapid Diagnostic Tests and Antimicrobial Stewardship Programs for the Management of Bloodstream Infection: What Is Their Relative Contribution to Improving Clinical Outcomes? A Systematic Review and Network Meta-analysis. Clin Infect Dis 79:502–515. https://doi.org/10.1093/cid/ciae234 10.1093/cid/ciae234 PMC11327801 38676943

[43] Satlin MJ, Chen L, Gomez-Simmonds A et al (2022) Impact of a Rapid Molecular Test for Klebsiella pneumoniae Carbapenemase and Ceftazidime-Avibactam Use on Outcomes After Bacteremia Caused by Carbapenem-Resistant Enterobacterales. Clin Infect Dis 75:2066–2075. 10.1093/cid/ciac354 35522019 10.1093/cid/ciac354 PMC10200298

[44] Wang Y, Lindsley K, Bleak TC et al (2025) Performance of molecular tests for diagnosis of bloodstream infections in the clinical setting: a systematic literature review and meta-analysis. Clin Microbiol Infect 31:360–372. 10.1016/j.cmi.2024.12.007 39672467 10.1016/j.cmi.2024.12.007

[45] Yee R, Dien Bard J, Simner PJ (2021) The Genotype-to-Phenotype Dilemma: How Should Laboratories Approach Discordant Susceptibility Results? J Clin Microbiol. 10.1128/JCM.00138-20 33441396 10.1128/JCM.00138-20 PMC8316082

[46] Perry JD (2017) A Decade of Development of Chromogenic Culture Media for Clinical Microbiology in an Era of Molecular Diagnostics. Clin Microbiol Rev 30:449–479. 10.1128/CMR.00097-16 28122803 10.1128/CMR.00097-16 PMC5355637

[47] Sherry NL, Lee JYH, Giulieri SG et al (2025) Genomics for antimicrobial resistance—progress and future directions. Antimicrobial Agents and Chemotherapy 69:e01082-01024. 10.1128/aac.01082-24 40227048 10.1128/aac.01082-24 PMC12057382

[48] Bortolaia V, Kaas RS, Ruppe E et al (2020) ResFinder 4.0 for predictions of phenotypes from genotypes. J Antimicrob Chemother 75:3491–3500. 10.1093/jac/dkaa345 32780112 10.1093/jac/dkaa345 PMC7662176

[49] Feldgarden M, Brover V, Gonzalez-Escalona N et al (2021) AMRFinderPlus and the Reference Gene Catalog facilitate examination of the genomic links among antimicrobial resistance, stress response, and virulence. Sci Rep 11:12728. 10.1038/s41598-021-91456-0 34135355 10.1038/s41598-021-91456-0 PMC8208984

[50] Pham NP, Gingras H, Godin C et al (2024) Holistic understanding of trimethoprim resistance in Streptococcus pneumoniae using an integrative approach of genome-wide association study, resistance reconstruction, and machine learning. mBio 15:e0136024. 10.1128/mbio.01360-24 39120145 10.1128/mbio.01360-24 PMC11389379

[51] Ma KC, Mortimer TD, Duckett MA et al (2020) Increased power from conditional bacterial genome-wide association identifies macrolide resistance mutations in Neisseria gonorrhoeae. Nat Commun 11:5374. 10.1038/s41467-020-19250-6 33097713 10.1038/s41467-020-19250-6 PMC7584619

[52] Farhat MR, Freschi L, Calderon R et al (2019) GWAS for quantitative resistance phenotypes in Mycobacterium tuberculosis reveals resistance genes and regulatory regions. Nat Commun 10:2128. 10.1038/s41467-019-10110-6 31086182 10.1038/s41467-019-10110-6 PMC6513847

[53] Biggel M, Johler S, Roloff T et al (2023) PorinPredict: In Silico Identification of OprD Loss from WGS Data for Improved Genotype-Phenotype Predictions of P. aeruginosa Carbapenem Resistance. Microbiol Spectr 11:e0358822. 10.1128/spectrum.03588-22 36715510 10.1128/spectrum.03588-22 PMC10100854

[54] Cintron M, Clark B, Miranda E, Babady NE (2025) Utility of digital images captured after 4 h of incubation on a microbiology laboratory automation system in guiding the work-up of subcultures from positive blood cultures. J Clin Microbiol 63:e0132024. 10.1128/jcm.01320-24 39704521 10.1128/jcm.01320-24 PMC11837544

[55] Cherkaoui A, Renzi G, Tittel-Elmer M, Schrenzel J (2025) Evaluation of PhenoMATRIX(R) PLUS for the culture-based rectal screening of CPOs, ESBL‑E, Acinetobacter, and VRE carriage. Front Microbiol 16:1624411. 10.3389/fmicb.2025.1624411 40687850 10.3389/fmicb.2025.1624411 PMC12271094

[56] McElvania E, Mindel S, Lemstra J et al (2024) Automated detection of methicillin-resistant Staphylococcus aureus with the MRSA CHROM imaging application on BD Kiestra Total Lab Automation System. J Clin Microbiol 62:e0144523. 10.1128/jcm.01445-23 38557148 10.1128/jcm.01445-23 PMC11077980

[57] Giske CG, Bressan M, Fiechter F et al (2024) GPT-4-based AI agents-the new expert system for detection of antimicrobial resistance mechanisms? J Clin Microbiol 62:e0068924. 10.1128/jcm.00689-24 39417635 10.1128/jcm.00689-24 PMC11559085

[58] Weis C, Cuenod A, Rieck B et al (2022) Direct antimicrobial resistance prediction from clinical MALDI-TOF mass spectra using machine learning. Nat Med 28:164–174. 10.1038/s41591-021-01619-9 35013613 10.1038/s41591-021-01619-9

[59] Nguyen HA, Peleg AY, Song J et al (2024) Predicting Pseudomonas aeruginosa drug resistance using artificial intelligence and clinical MALDI-TOF mass spectra. mSystems 9:e0078924. 10.1128/msystems.00789-24 39150244 10.1128/msystems.00789-24 PMC11406958

[60] Gato E, Arroyo MJ, Mendez G et al. (2023) Direct Detection of Carbapenemase-Producing Klebsiella pneumoniae by MALDI-TOF Analysis of Full Spectra Applying Machine Learning. J Clin Microbiol 61:e0175122. https://doi.org/10.1128/jcm.01751-22 10.1128/jcm.01751-22 PMC10281162 37199638

